# Unusual Etiology of Chronic Posterior Leg Pain in a Running Athlete: Could It Be a Schwannoma? A Case Report and Review of the Literature

**DOI:** 10.1155/2019/2307153

**Published:** 2019-11-12

**Authors:** Naoufal Elghoul, Omar Zaddoug, Mohammed Tbouda, Mohammed Benchakroun, Abdeloihab Jaafar

**Affiliations:** ^1^Department of Orthopedic Surgery and Traumatology, Military Hospital Mohammed V (HMIMV), Faculty of Medicine and Pharmacy, Mohammed V University of Rabat, BP 10100, Morocco; ^2^Department of Anatomy Pathology, Military Hospital Mohammed V (HMIMV), Faculty of Medicine and Pharmacy, Mohammed V University of Rabat, BP 10100, Morocco

## Abstract

Schwannomas represent only 5% of all soft tissue tumors. As a variant of this tumor, the plexiform schwannoma is rare accounting for less than 5% of all schwannomas. Herein, we report a rare case of a 49-year-old athlete who suffered from a pain in the posterior aspect of the right leg one year before his presentation. Initially, a radiograph of his right leg showed no abnormality, and so, the emergency physician discharged him on analgesics and anti-inflammatory medications, and rest was advised. The persistent pain obliged the patient to consult our orthopedic department. On examination, we found a firm mass in the proximal medial aspect of his right leg. The neurovascular exam was normal. Sonography of the leg was not conclusive. Therefore, magnetic resonance imaging was performed, and a hemangioma or schwannoma was suspected. The patient underwent surgery in which the entire tumor mass was shelled out in one piece with no damage. The histopathological finding was concomitant with a plexiform schwannoma. Follow-up evaluation, sixteen months later, showed no evidence of recurrence, and the patient has regained his previous level of sportive activities. So, given the case described here, despite the rarity of the schwannoma, it should be taken into consideration as a possible diagnosis in such situation to promote early diagnosis and appropriate treatment.

## 1. Introduction

Schwannomas, also known as neurilemomas, are benign peripheral nerve sheath tumors composed exclusively of Schwann cells; they account for only 5% of all soft tissue tumors [1, 2]. These lesions are commonly encountered in the head and neck region [3]. In the lower limb, they occur rarely in collateral branches of a nerve [4–7]. Furthermore, as a variant of this tumor, the plexiform schwannoma is rare which accounts for less than 5% of all schwannomas and it is difficult to diagnose [8]. Moreover, it is often misdiagnosed due to lack of awareness and often confused with other common lesions like lipoma and fibroma of the ganglion [9]. However, its diagnosis can be suspected on sonography, computed tomography, and magnetic resonance imaging examination, and it is confirmed by pathological analysis of the operative specimen [10, 11]. To the best of our knowledge, the case presented here is among the extremely rare cases of posterior leg pain in running athletes due to an atypical localization of a rare schwannoma.

## 2. Case Presentation

A 49-year-old athlete suffered from pain localized in the posterior aspect of the right leg since one year, with no reported history of trauma or prick injury. He consulted initially an emergency physician who has requested a radiograph of the right leg which showed no pathology, and so, the patient was discharged on analgesics and anti-inflammatory medications, and rest was advised.

At home, the patient did well, but he continued to experience intermittent pain in the same localization, which prevented him from carrying out his activities. One month later, he consulted our orthopedic department for further evaluations. On admission, he reported a localized moderate pain in the proximal medial side of his right leg, burning in type with no radiation, and it increased slightly with active and passive dorsiflexion of the foot. In this localization, the clinical examination found a firm mass measuring 2.5 cm/2 cm mobile transversely and slightly longitudinally. The neurovascular examination was normal.

**Figure 1:**
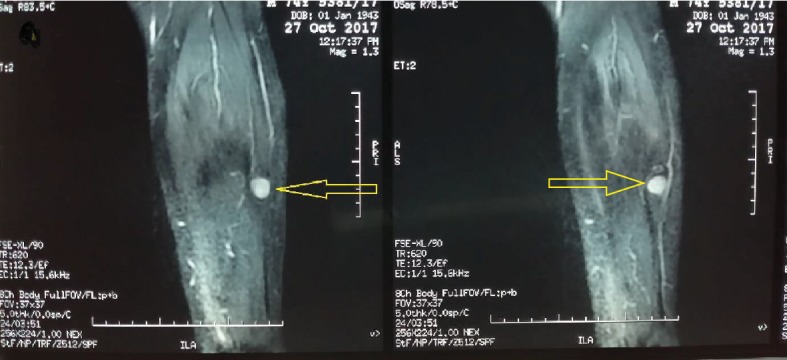
MRI T1 fat suppression showed the tumor mass (arrows).

**Figure 2:**
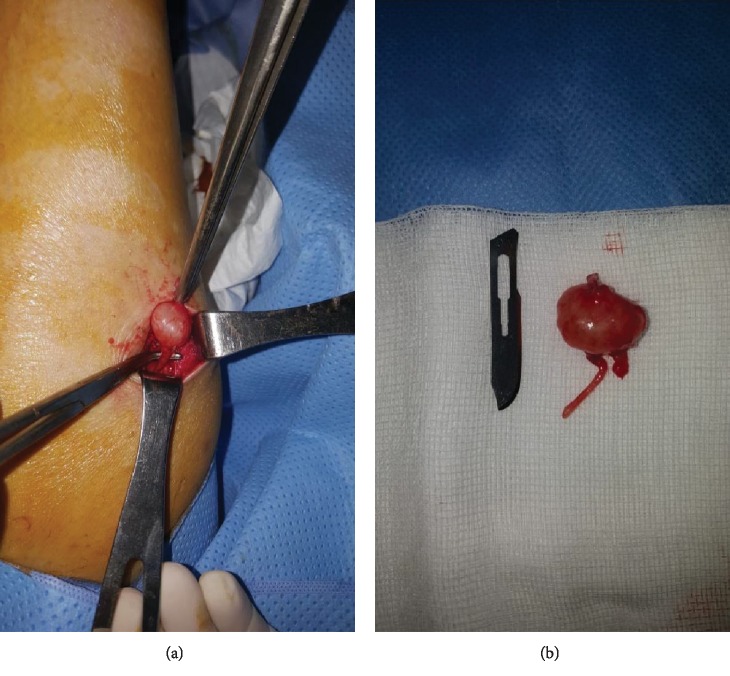
(a, b) Clinical aspect of the tumor mass.

**Figure 3:**
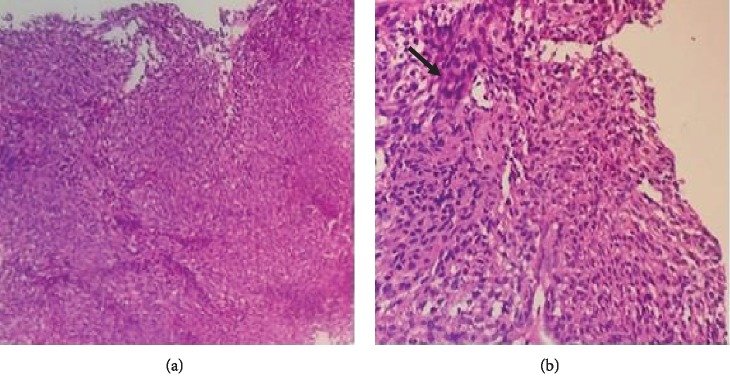
Histological section at low (a) and medium (b) magnification showing tumor proliferation. Note the Verocay bodies (black arrow) (HE ×100; HE ×200).

**Figure 4:**
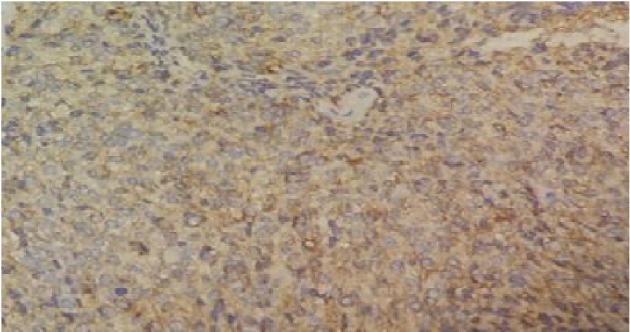
Immunohistochemical study showed a positive staining of the tumor cells by the anti-PS 100.

On investigation, he had a hemoglobin of 12 g/dl and normal white blood cell counts, platelets, and D-dimer. Since the sonography of the leg was not conclusive, we performed, a few days later, a magnetic resonance imaging of the leg that was in favor of hemangioma or schwannoma (Figure 1), prompting the patient to undergo surgery. Thus, under spinal anesthesia along with tourniquet control, the entire tumor mass was shelled out carefully in one piece with no damage (Figure 2) and was sent for histopathological examination. At the first day postoperatively, clinical assessment of the patient found no pain and the neurovascular examination was normal reason for which we discharged him on oral analgesics waiting for the histopathological result. Ten days later, the result was concomitant with plexiform schwannoma with no sign of degeneration (Figures 3 and 4).

At the last follow-up of sixteen months, he did not present any evidence of recurrent pain and he regained his previous level of sportive activities.

## 3. Discussion

For orthopedic surgeons taking care of athletes, chronic leg pain in athletes is a common complaint [12, 13]. The main etiologies of this pain include medial tibial stress syndrome, chronic exertional compartment syndrome, tibial stress fracture, popliteal artery entrapment syndrome, Achilles tightness, deep vein thrombosis, complex regional pain, and nerve entrapment [14]. Our case presented an extremely rare case of atypical localization of plexiform schwannoma which caused chronic leg pain. Schwannoma is a benign encapsulated tumor that originates from Schwann cells in the peripheral nervous system [15]. Diagnosis is habitually incidental [16, 17], not as in our case. It typically is painless, slow-growing, isolated, firm, and round soft tissue mass [2]. The lesion is often mobile in a transverse direction but not longitudinally. It is frequently misdiagnosed as a ganglion and may have a similar consistency [18, 19]. High-resolution ultrasonography, computed tomography, and magnetic resonance imaging had enhanced the preoperative diagnosis of nerve sheath tumors. However, the major limitation of high-resolution ultrasonography is operator dependence. For computed tomography, it is irradiation [20, 21]. For magnetic resonance imaging, it can be useful in delineating this lesion, but it may not be possible to distinguish neurilemoma from a neurofibroma or malignant peripheral nerve sheath tumor reason for which the pathological analysis of the operative specimen is necessary [22]. Most patients with a small solitary schwannoma are managed nonoperatively except if they manifest a progressive neurological deficit, pain, need for tissue diagnosis, or growth on serial imaging [23]. Surgically, schwannomas can be dissected carefully from the surrounding nerve while preserving the continuity of the nerve, in most cases, because those tumors repulse nerve fascicular groups without penetrating them [23]. Loss of function from smaller benign schwannomas is rare unless a prior biopsy had injured the involved nerve or an unsuccessful attempt at tumor removal had been performed. Its histology reveals spindle-shaped cells that are often palisading in a mixture of Antoni A and Antoni B patterns [24]. The Antoni A area is composed of spindle-shaped Schwann cells arranged in interlacing fascicles. There may be nuclear palisading. In between two compact rows of well-aligned nuclei, the cell processes form eosinophilic Verocay bodies. Mitotic figures may be present. The Antoni B area consists of a loose meshwork of gelatinous and microcystic tissue. Large, irregularly spaced, thick-walled blood vessels are noted in the Antoni B area. These may contain thrombus material in the lumina. Immunohistochemistry shows that S-100 stain was positive [25].

## 4. Conclusion

Although Schwannomas are uncommon, they should be considered as a possible cause of chronic leg pain. Its diagnosis is suggested by clinical and MRI examinations and is confirmed by histopathological analysis.
